# Brief online negative affect focused functional imagery training (FIT) improves four-week drinking outcomes in hazardous student drinkers: A pilot randomised controlled trial replication in South Africa

**DOI:** 10.1016/j.abrep.2024.100540

**Published:** 2024-03-19

**Authors:** Ruichong Shuai, Fatima Ahmed-Leitao, Jenny Bloom, Soraya Seedat, Lee Hogarth

**Affiliations:** a School of Psychology, University of Exeter, Exeter, United Kingdom; b Department of Psychiatry, Stellenbosch University, Private Bag X1, Matieland, 7602, Stellenbosch, South Africa

**Keywords:** Pilot RCT replication, Emotion regulation, Guided imagery, Negative affect drinking

## Abstract

**Background:**

Previous study has shown that functional imagery training (FIT) to utilise positive mental imagery in response to negative affect could improve alcohol-related outcomes. The current study aimed to replicate whether this negative affect focused FIT would improve alcohol-related outcomes in hazardous student drinkers in South Africa at four-week follow-up.

**Methods:**

50 hazardous student drinkers who reported drinking to cope with negative affect were randomised into two groups. The active group (n = 25) was trained online over two weeks to respond to personalised negative drinking triggers by retrieving a personalised adaptive strategy they might use to mitigate negative affect, whereas the control group (n = 25) received standard risk information about binge drinking. Outcome measures including alcohol consumption, drinking motives, anxiety and depression, self-efficacy and use of protective behavioural strategies were obtained at baseline and four-week follow-up.

**Results:**

FIT effects were revealed by three significant group-by-timepoint interactions in a per-protocol analysis: there was a significant decrease in depressive symptoms, drinking to cope and drinking for social reasons from baseline to follow-up in the active group, but not the control group. No effects were observed on alcohol consumption, self-efficacy, protective behaviour strategies and anxiety.

**Conclusions:**

Preliminary evidence supports that online negative affect focused FIT can improve depression as well as coping and social drinking motives in South African hazardous student drinkers who drank to cope, at four-week follow-up, suggesting that the principles of this FIT approach might be adapted and incorporated into a clinical intervention to test for efficacy in mitigating substance use problems.

## Highlights

• FIT trains positive mental imagery in response to negative affect.
• FIT improves depression and drinking for cope and social motives at four-week follow-up.
• FIT may build resilience to negative affect over time.
• The effect of FIT on outcomes was replicated cross culturally.

## 1 Introduction

The persistent high prevalence of excessive alcohol use is a well-documented issue in undergraduate populations across countries (Davoren et al., 2016, Hingson, Heeren, Winter, & Wechsler, 2005, Karam et al., 2007, Tarrant et al., 2019, Wicki et al., 2010). For example, one study in South Africa (SA) has found that out of 722 university students, 22.2 % was hazardous or harmful alcohol users (Pengpid et al., 2013). Despite college students drinking for various reasons, drinking to cope with negative affect has been found to uniquely confer a greater risk of developing alcohol-related problems, including alcohol dependence (Cooper et al., 1995, Kuntsche et al., 2005, Merrill et al., 2014). This unique risk has also been observed in university students in SA (Peltzer, 2003). Furthermore, it has been shown that drinking to cope with negative affect mediates hazardous drinking in college students who have experienced psychiatric symptoms, abuse or trauma (Bravo and Pearson, 2017, Goldstein et al., 2010, Shuai et al., 2022, Villarosa et al., 2018), and mediates the link between childhood abuse and alcohol problems in SA adolescents (Hogarth et al., 2019). The implication is that training adaptive strategies to deal with negative affect may be beneficial in these high-risk groups to mitigate alcohol problems (Aurora and Klanecky, 2016, Wahesh et al., 2020). One effective approach is an episodic future thinking (EFT) intervention, which trains individuals to imagine their best possible future self or positive future events/goals, and has been shown to improve anxiety and depressive symptoms (Renner et al., 2014, Weßlau and Steil, 2014), enhance positive mood (Schubert et al., 2020), reduce alcohol demand and consumption in college students (Bulley and Gullo, 2017, Voss et al., 2022) and in alcohol dependent individuals (Athamneh et al., 2022, Patel and Amlung, 2020, Snider et al., 2016).

Functional imagery training (FIT) is another imagery-based intervention similar to EFT. FIT trains individuals to embed retrieval of imagery about the positive outcomes (e.g., feeling good about themselves) resulting from behaviour change into daily routine (such as when brushing teeth or walking upstairs), in the hope that the retrieval routine will promote behaviour change. For instance, training individuals such that when they are in risky situations (e.g., craving food) to retrieve imagery about the benefits of abstinence has been shown to be effective in reducing snacking (Andrade et al., 2016) and maintaining weight loss over time in overweight individuals (Solbrig et al., 2019). Furthermore, therapeutic approaches akin to FIT have been shown to improve emotional self-regulation and reduce self-harm (Di Simplicio et al., 2020), suggesting that an FIT intervention might have efficacy in reducing reactivity to negative affect and in turn, negative affect motivated drinking behaviour (May et al., 2015). Emerging evidence supporting this has come from two recent studies, which shows that an online-delivered negative affect focused FIT intervention had an immediate effect on attenuating noise stress-induced alcohol-seeking in the lab (Bakou et al., 2021) and improved alcohol-related outcomes in real-life settings at two-week follow-up in a randomised controlled trial with hazardous student drinkers recruited from the UK (Shuai et al., 2021). The therapeutic objective of FIT in this trial was to break the link between personalised negative affect triggers and drinking behaviour by training routine retrieval of alternative adaptive strategies tailored to individuals in response to those triggers, in the hope of reducing drinking outcomes. Relative to baseline, two weeks of online training on this negative affect focused FIT intervention increased self-reported self-efficacy of control over negative affect drinking, increased self-efficacy of control over consumption, and reduced motives to drink for social reasons, compared to a generic alcohol health information control group. However, there was no effect of FIT on self-reported drinking to cope with negative affect – the core construct that the intervention was specifically designed to modify – and there was no effect on alcohol consumption.

The purpose of the present study was to replicate the previous study conducted in the UK (Shuai et al., 2021) with a 4-week follow-up period to assess longer-term outcomes, and with a sample of hazardous student drinkers who drink to cope, recruited within South Africa, to test the cross-cultural replicability of the effects of FIT. The study protocol was identical to the previous UK study and was registered with the Pan African Clinical Trial Registry (https://www.pactr.org) (PACTR202011530106411). Apart from the sample, there was one major procedural modification. During the first two weeks, participants received their initial training video online and then received daily emails or texts to promote and quantify engagement with the FIT or control intervention as before. However, the outcome measures were not recorded at the end of these two weeks as before, to avoid potential anchoring of responses (Gehlbach & Barge, 2012) which may be more common in online surveys (Huang et al., 2015, Meade and Craig, 2012). Instead, the current trial allowed a further two-week period in which there was no engagement with participants before the outcome measures were recorded (at the final four-week timepoint). This modification was to test the durability of longer-term effects. In a previous FIT intervention for weight loss (Solbrig et al., 2019), it was found that three months after training-related contact from the experimenter had ended, participants in the FIT intervention group showed further weight loss, compared to controls, suggesting they had adopted the technique into their daily life. In the current study, it was hoped that the additional two-week period after training-related engagement had ended would allow greater therapeutic effects to emerge, at the terminal four-week timepoint, suggesting adoption of the technique into daily life.

## 2 Methods

### 2.1 Participants

**Fig. 1:**
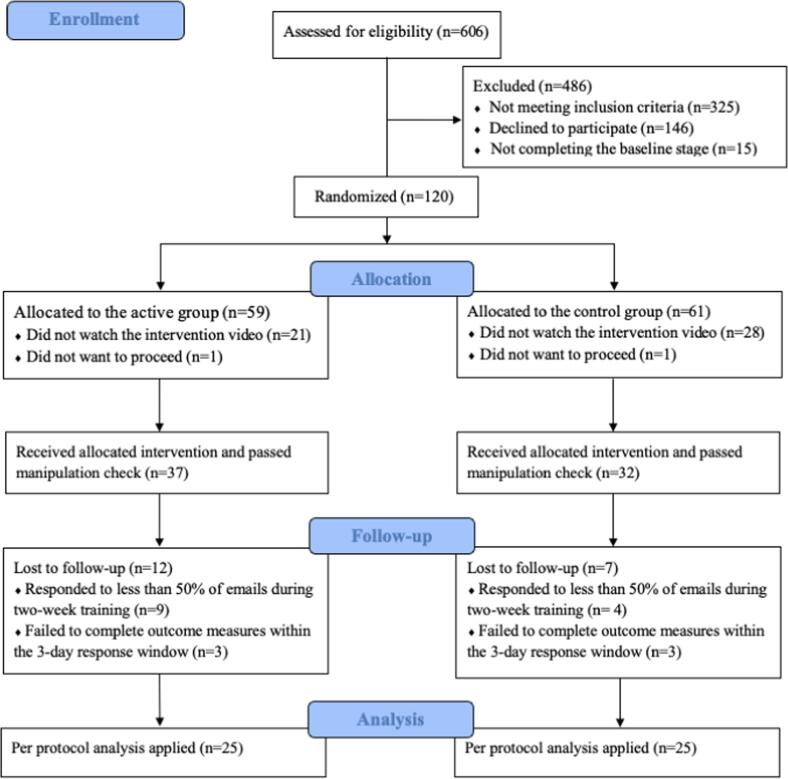
CONSORT diagram displaying the progress and attrition through the intervention and follow.

Participants were recruited using convenience sampling from Stellenbosch University via email invitation and screened in the study. Of 606 participants initially screened, 50 consented and completed the whole study and were included in the final analysis. Participant inclusion/exclusion at each stage is detailed in Fig. 1. The target sample size after exclusion was sought to be close to the previous study carried out in the UK (N = 52) (Shuai et al., 2021) and other previous proof-of-concept FIT trials (N = 24–45) (Andrade et al., 2016, Di Simplicio et al., 2020, Rhodes et al., 2018). The sample size was constrained by the time and resources available to conduct the study in SA. Our primary aim was to determine whether the previous medium to large manipulation effects (η_p_^2^ varying from 0.080 to 0.462) obtained in the UK sample would replicate in an SA sample of comparable size. Post-hoc power analysis was calculated using G*Power 3.1.9.7 (Faul et al., 2007) which showed that the current sample size of 50 achieved 99 % power for detecting a medium effect size for an interaction (f = 0.25, (Cohen, 2013) in a mixed 2-way ANOVA at a significance criterion of α = 0.05.

### 2.2 Materials

**Screening measures.** Initial screening followed exactly the same procedure as the previous study conducted in the UK. To participate, participants had to be aged between 18 and 25 years old and be a student. They had to score ≥ 8 on the 10-item Alcohol Use Disorder Identification Test (AUDIT) indicating that they were hazardous drinkers (or higher risk drinkers if > 15, (DeMartini & Carey, 2009). Finally, they had to endorse ≥ 5 items on the 35-item Drinking to Cope Checklist (DTCC) (e.g., ‘I am more likely to drink when I feel stressed’) indicating they had at least five negative affect drinking triggers (Shuai et al., 2021).

**Intervention.** Intervention materials were adapted from the previous UK study. Both intervention videos (active or control) were PowerPoint presentations containing text and images, with the text read out by the same female voice while it was presented on screen. The active intervention video was 8 minutes and contained 13 pages, consisting of three major parts: 1) information about participants’ risk of developing future alcohol use problems because they reported drinking to cope; 2) a personalised list of the negative affective states that they had endorsed as motivating their drinking on the DTCC, and 3) guided imagery training on vividly imaging what their best self would do in response to their negative affective drinking triggers with adaptive strategies (example video available at YouTube link: https://youtu.be/xbhTxFCcRjY). Training ended by participants being told that they should practice this reactive imagery technique over the next four weeks. The control intervention video was 4 minutes and contained 13 pages. The content summarised the binge drinking risk information derived from the US National Institute on Alcohol Abuse and Alcoholism (NIAAA, 2011) College Drinking Factsheet (see also YouTube link for video: https://youtu.be/08ra9hBqcf4).

**Baseline and outcome measures.** The same questionnaire set as in the previous UK study was completed at baseline and four-week follow-up. Cronbach’s α was calculated for scales and most of them had good internal consistency (Tavakol & Dennick, 2011, see Table 2 for details).

The Daily Drinking Questionnaire – Revised (hereafter ‘Alcohol Consumption’) was used to measure drink type and volume consumed in each of the past 14 days, rescored as sum of alcohol units consumed over the past 14 days (Kruse et al., 2005).

Protective Behaviour Strategies Scale-Revised (hereafter ‘Protective Behaviours’) was used to assess the use of various strategies to control drinking behaviour in the past two weeks with 20 items (e.g., “Determine not to exceed a set number of drinks”), endorsed on a 6-point Likert scale ranging from 1 (‘Never’) to 6 (‘Always’), averaged to yield a single score (Treloar et al., 2015).

Controlled Drinking Self-Efficacy Scale (hereafter ‘Self-efficacy’) contains 20 items, which assessed participants’ confidence in controlling drinking in the next six months for various risk scenarios, endorsed on a scale ranging from 0 (‘not confident’) to 10 (‘very confident’) (Kemp et al., 2007, Sitharthan et al., 2003). The scale contains 4 subscales which evaluate different risk scenarios: negative affect (‘when you are irritated’), frequency of drinking (‘Can you stop yourself from drinking alcohol at least one day a week’), positive mood/social context (‘when you want to feel more confident’), and consumption quantity (‘Can you make sure that you do not have more than three drinks on any time that you have a drink’).

The Drinking Motives Questionnaire – Revised is a five-dimensional questionnaire measuring drinking motives in the past two weeks, which will hereafter be labelled as ‘Drink Motives-anxiety’ (e.g., ‘to relax’), ‘Drink Motives-depression’ (e.g., ‘to numb my pain’), ‘Drink Motives-enhancement’ (e.g., ‘to get a high’), ‘Drink Motives-conformity’ (e.g., ‘to be liked’) and ‘Drink Motives-social’ (e.g., ‘as a way to celebrate’), endorsed on a scale ranging from 0 (‘never’) to 10 (‘always’) (Grant et al., 2007). Drink Motives-anxiety and Drink Motives-depression were highly correlated at baseline, *r* = 0.61, *p* <.001, and at follow-up, *r* = 0.72, *p* <.001, and so were averaged into one single ‘Drink Motives-coping’ score for analysis.

The Generalised Anxiety Disorder Questionnaire (hereafter ‘Anxiety’) containing 7 items was used to measure the symptoms of generalised anxiety disorder (e.g., ‘feeling nervous, anxious or on edge’) in the past two weeks. The score on each item ranges from 0 (‘Not at all’) to 3 (‘Nearly every day’). The total score can range from 0 to 21, with a score of 10 as the cut-off point for moderate anxiety (Spitzer et al., 2006).

The Patient Health Questionnaire Depression Scale (hereafter ‘Depression’) containing 8 items was used to measure symptoms of current depression (e.g., ‘little interest or pleasures in doing things’) in the past two weeks. The score on each item ranges from 0 (‘Not at all’) to 3 (‘Nearly every day’). The total score can range from 0 to 24, with a score of 10 as the cut-off point for moderate depression (Kroenke et al., 2009).

### 2.3 Procedure

Ethical approval was obtained from Stellenbosch University’s Health Research Ethics Committee (Ethics reference no.: N20/04/051). The study lasted for a 3-month period from the beginning of September to beginning of December 2021. The whole procedure was conducted by research team at Stellenbosch University, with regular assistance and check-in from the first author of this paper. Undergraduates who expressed interest in participating were sent a survey link containing the informed consent form and screening questionnaires. Eligible participants were then sent the baseline questionnaires, and those who completed it were randomised to the active or control group. Block randomization was employed using a computer random number generator, with a fixed block size of 4. Each group was sent a video to watch which contained the intervention information within two days of randomisation. All the surveys and intervention videos were set up and administered on Qualtrics survey engine, adapted from the previous UK study. Upon completion of watching the intervention video, all participants went through a manipulation check consisting of five questions to assess their understanding of the video content, with above 80% accuracy progressing to the follow-up stage. Over the first two-week follow-up period, participants in both groups were emailed or messaged daily and asked to report their engagement with the intervention material in the prior 24-hour period. The active group were asked ‘Did you practice the imagery technique yesterday the [date inserted]?’, answered on a yes/no scale, and ‘if so, how many times?’. The control group were asked ‘Did you think about the health information about binge drinking yesterday the [date inserted]?’, answered on a yes/no scale, and ‘if so, how many times?’. The average number of times that participants engaged with the intervention (averaged across available email responses) quantified engagement with the intervention. Participants who did not respond to three consecutive, or seven in total, follow-up emails, were withdrawn (see Fig. 1). At the end of the first two-week training period, participants were encouraged to continue to utilise what they had learned during the intervention phase. Over the second two-week follow-up period, participants had no contact with the researchers and received no assessment of their use of intervention technique. At the four-week follow-up timepoint (i.e., four weeks after exposure to the active or control intervention video), participants completed the outcome questionnaires (for comparison with baseline). Participants were debriefed and received ZAR250 upon completion of the study.

### 2.4 Analytical plan

Due to high attrition, the current study reported a similar per protocol analysis as in the previous UK study, which only included participants who completed the four-week follow-up period (see Fig. 1: active n = 25, control n = 25). IBM SPSS Statistics 28 was used for data analysis. Assumption tests showed that normal distribution and equal variance were violated for some outcome measures. However, ANOVA is regarded as being robust to non-normal distribution (Blanca et al., 2017) and variance heterogeneity when sample sizes are equal (Glass et al., 1972). Analytical procedures in the current study were identical to the previous UK study, in which each questionnaire scale (total score or subscales) was entered into a mixed ANOVA with the variable group (active, control) as the between-subjects factor and timepoint (baseline, follow-up) as the within-subjects factor. Significant interactions were followed up with specific contrasts (ANOVAs) testing the timepoint effect in each group and the group effect at each timepoint.

## 3 Results

### 3.1 Participants and exclusions

**Fig. 2:**
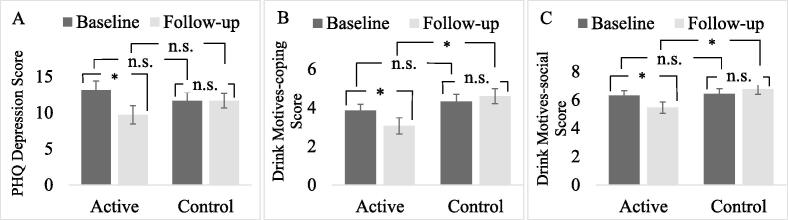
Outcome measures that changed from baseline to follow-up timepoints differentially between the active and control group. Fig. 2 shows the three outcome measures which showed changes from baseline to follow-up timepoints differentially between the active and control group (i.e., a significant group by timepoint interaction): (A) Depression, (B) Drink Motives-coping and (C) Drink Motives-social. The lines describe the specific contrasts. * = p < .05, n.s.= not significant.

Baseline characteristics were mostly matched between the active and control groups, except that the control group endorsed more negative affect drinking triggers than the active group (see Table 1). This construct of using alcohol to cope with negative affect was further assessed by the Drink Motives-Coping item from Drinking Motives Questionnaire Revised (Grant et al., 2007) as an outcome measure at both baseline and four-week follow-up and reported in the text below (also see Table 2 and Fig. 2).

**Table 1:** Mean (standard deviation, range) of questionnaire data reported by the active and control groups.

		Groups		Test statistics		
		Active (n = 25)	Control (n = 25)			
**Independent *t*-test**				t	df	*p*
Age in years		20.60 (1.58, 18–24)	21.04 (1.88, 18–25)	0.90	48	0.375
AUDIT score		14.64 (6.10, 8–28)	14.68 (5.44, 8–27)	0.02	48	0.981
DTCC items endorsed (%)		36.91 (18.64, 14.29–77.14)	50.40 (19.08, 17.14–80)	2.53	48	**0.015**
Average number of times of practicing/thinking about the intervention in the previous day		1.28 (1.00, 0–4.62)	1.10 (0.73, 0–2.80)	−0.73	48	0.469

**Independent-samples Mann-Whitney *U* test**				U	z	*p*
Email response rate during the follow-up (%)		94.57 (5.56, 85.71–100)	89.43 (12.22, 64.29–100)	360.50	0.99	0.324

**Chi-square test**				*X^2^*	N	*p*
Gender ratio (M/F)		4/21	5/20	-	-	1.00^a^
Drinking frequency	Daily	0	0	2.12	50	.244^b^
	Weekly	52 %	72 %			
	Monthly	48 %	28 %			

*note.* AUDIT = Alcohol Use Disorder Identification Test. DTCC = Drinking to Cope Checklist. All p-values were calculated for two-sided tests. Significant *p*-value is emboldened. a – Fisher’s Exact Significance Test (>20 % of expected cell counts are less than 5). b – Pearson Chi-Square Exact Significance Test.

**Table 2:** Mean (standard deviation, range) of outcome measures at baseline and four-week follow-up timepoints for the active (FIT) and control group.

	Mean (standard deviation, range)				ANOVA statistics					
	Active group (n = 25)		Control group (n = 25)		Main effect of group		Main effect of timepoint		Group x timepoint interaction	
Questionnaire (α)	Baseline	Follow-up	Baseline	Follow-up	F	η_p_^2^	F	η_p_^2^	F	η_p_^2^
Alcohol Consumption (0.73)	27.52 (24.24, 0–88)	21.56 (17.23, 0–66.50)	38.07 (64.99, 0–335)	31.50 (39.57, 0–182.60)	0.93	0.019	1.87	0.037	0.004	0
Protective Behaviours (0.83)	3.79 (0.93, 1–5.40)	4.02 (1.16, 1–5.45)	3.79 (0.69, 2.80–6)	3.94 (0.88, 1–5.35)	0.03	0.001	2.04	0.041	0.11	0.002
Self-efficacy Negative Affect (0.83)	5.08 (1.81, 0.89–8.78)	5.69 (2.33, 0–8.89)	4.92 (1.95, 1.33–9)	5.24 (2.04, 0–8.67)	0.47	0.010	1.66	0.033	0.17	0.004
Self-efficacy Frequency of Drinking (0.85)	7.76 (1.58, 4–9)	7.77 (2.19, 0–9)	7.25 (2.33, 2.33–9)	8.47 (1.15, 3.67–9)	0.05	0.001	4.01	0.077	3.83	0.074
Self-efficacy Positive Mood /Social Context (0.57)	3.80 (1.56, 1.17–7.83)	4.73 (1.76, 0.33–8.67)	4.59 (1.70, 1.17–9)	4.16 (1.97, 0–8)	0.10	0.002	0.52	0.011	3.91	0.075
Self-efficacy Consumption Quantity (0.66)	4.46 (2.02, 0–8)	4.28 (3.16, 0–9)	4.04 (2.50, 0.50–9)	4.50 (2.96, 0–9)	0.03	0.001	0.10	0.002	0.53	0.011
Drink Motives-social (0.80)	6.31 (1.66, 2.40–9)	5.46 (1.98, 1–8.60)	6.42 (1.89, 1–9)	6.76 (1.82, 0–9)	2.35	0.047	1.17	0.024	6.12*	0.113
Drink Motives-coping (0.89)	3.85 (1.62, 1.40–8)	3.06 (2.08, 0–8.54)	4.31 (1.89, 1.38–8.88)	4.60 (1.92, 0.25–7.24)	4.36*	0.083	1.17	0.024	5.30*	0.099
Drink Motives-enhancement (0.76)	4.68 (1.79, 0.80–7.60)	3.90 (2.46, 0–9)	5.22 (2.02, 1.60–9)	5.08 (2.29, 0–9)	2.68	0.053	2.15	0.043	1.06	0.022
Drink Motives-conformity (0.83)	2.04 (2.19, 0–7)	1.60 (2.17, 0–7.40)	2.24 (1.84, 0–6)	2.23 (1.89, 0–7)	0.63	0.013	0.89	0.018	0.83	0.017
Anxiety (0.91)	11.00 (6.13, 0–21)	9.28 (6.45, 0–20)	10.56 (5.72, 1–21)	10.56 (5.99, 1–21)	0.08	0.002	1.09	0.022	1.09	0.022
Depression (0.89)	13.12 (6.37, 2–21)	9.72 (6.33, 0–22)	11.68 (5.63, 2–23)	11.68 (5.16, 3–21)	0.03	0.001	4.34*	0.083	4.34*	0.083

*note.* The right-hand columns report statistics from mixed ANOVAs testing the main effects and interaction of group and timepoint for each outcome measure (significant F values are emboldened, * = *p* <.05). A significant interaction reveals an intervention effect. Cronbach’s α reliability score for each outcome measure is reported in brackets following the name of the measure. Alcohol Consumption = sum of units consumed over a four-week period time, recorded by Daily Drinking Questionnaire Revised. Protective Behaviours = Protective Behaviour Strategies Scale – Revised. Controlled Drinking Self-Efficacy Scale is reported with four subscales, Self-efficacy Negative Affect, Self-efficacy Frequency of Drinking, Self-efficacy Positive Mood/Social Context, and Self-efficacy Consumption Quantity. Drinking Motives Questionnaire – Revised is reported with four subscales, Drink Motives-social, Drink Motives-coping, Drink Motives-enhancement and Drink Motives-conformity. Anxiety = Generalised Anxiety Disorder Questionnaire. Depression = Patient Health Questionnaire Depression Scale.

### 3.2 Outcome measures

Table 2 presents the absolute scores for each outcome in the order they were collected, plus the results of the group by time ANOVA applied to that data. Fig. 2 presents those outcomes which showed a significant group by timepoint interaction, for emphasis.

Regarding the Depression outcome, as shown in Table 2 (and Fig. 2), there were a significant group by timepoint interaction and a main effect of timepoint, but no main effect of group. Specific contrasts revealed that Depression reduced significantly from baseline to follow-up in the active group, *F* (1, 24) = 6.46, *p* =.018, η_p_^2^ = 0.212, but not the control group, *F* (1, 24) = 0, *p* = 1.000, η_p_^2^ = 0. However, there was no significant group difference at baseline, *F* (1, 48) = 0.72, *p* =.401, η_p_^2^ = 0.015, or follow-up, *F* (1, 48) = 1.44, *p* =.236, η_p_^2^ = 0.029.

Regarding the Drink Motives-coping outcome, as shown in Table 2 (and Fig. 2), there were a significant group by timepoint interaction and a main effect of group, but no main effect of timepoint. Specific contrasts revealed that Drink Motives-coping reduced significantly from baseline to follow-up in the active group, *F* (1, 24) = 4.46, *p* =.045, η_p_^2^ = 0.157, but not the control group, *F* (1, 24) = 1.04, *p* =.319, η_p_^2^ = 0.041. Furthermore, there was a significant group difference for Drink Motives-coping at follow-up, *F* (1, 48) = 7.38, *p* =.009, η_p_^2^ = 0.133, but not at baseline, *F* (1, 48) = 0.86, *p* =.358, η_p_^2^ = 0.018.

Regarding the Drink Motives-social outcome, as shown in Table 2 (and Fig. 2), there was a significant group by timepoint interaction and no main effects of either variable. Specific contrasts revealed that Drink Motives-social reduced significantly from baseline to follow-up in the active group, *F* (1, 24) = 4.81, *p* =.038, η_p_^2^ = 0.167, but not the control group, *F* (1, 24) = 1.41, *p* =.246, η_p_^2^ = 0.056. Furthermore, there was a significant group difference for Drink Motives-social at follow-up, *F* (1, 48) = 5.87, *p* =.019, η_p_^2^ = 0.109, but not at baseline, *F* (1, 48) = 0.05, *p* =.825, η_p_^2^ = 0.001.

Notably, no significant results were obtained after Bonferroni correction was applied for multiple testing in outcome measures (discussed as a limitation below).

### 3.3 Exploratory correlational analysis

To explore therapeutic mechanisms, a bivariate Pearson correlation matrix with the entire analytic sample tested all inter-correlations between the change scores from baseline to follow-up timepoints for the outcome measures which showed a significant intervention effect. The results showed that the reduction in Depression was significantly correlated with the reduction in Drink Motives-coping, *r* = 0.51, *p* <.001, but not significantly with reduction in Drink Motives-social, *r* = 0.28, *p* =.051, providing insight into candidate mechanisms of the therapeutic effect.

## 4 Discussion

The current study was a replication of the previous UK study to test whether a FIT intervention linking experience of negative affect to retrieval of mental imagery of adaptive strategies, would reduce psychiatric symptom severity (depression and anxiety), alcohol consumption, and drinking-related measures (drinking motives, self-efficacy, protective behaviour strategies), in hazardous drinking students who reported drinking to cope with negative affect in SA, at a four-week follow-up timepoint. The study found that compared to the control group, participants in the active (FIT) group showed greater improvements in three outcome measures. First, the active group showed a significant decline in depressive symptoms at the four-week follow-up, reporting fewer symptoms such as ‘Feeling down, depressed, or hopeless’. Secondly, the active group showed a significant reduction in drinking to cope, perceiving their drinking as less motivated by reasons like ‘Because it helps me when I am feeling depressed”. Thirdly, the active group showed a significant reduction in social drinking motives, perceiving their drinking as less motivated by such items as: ‘To be sociable’. These findings extend the previous UK trial (Shuai et al., 2021), showing that two weeks of online training to respond to negative affect drinking triggers by retrieving future adaptive strategies has efficacy in improving drinking related outcomes in hazardous, negative affect student drinkers from SA and over an extended four-week follow-up period. Furthermore, relative to the previous UK study, the current study has found that reduction in depression was correlated with reduction in drinking to cope over the four-week follow-up. This finding is encouraging, as the intervention was specifically designed to mitigate negative affect motivated drinking and may shed some light on the potential mechanism of this FIT intervention (details discussed below).

In direct comparison to the previous UK study, the current study replicated only one finding, namely significant reduction in belief of drinking for socialising in the active group from baseline to four-week follow-up, compared to the control group. As hazardous drinking in undergraduate students is often associated with social occasions (Christiansen et al., 2002, Lindgren et al., 2013), it might be anticipated that drinking related to social reasons could be more sensitive to alcohol interventions and thus, a consistent intervention effect has been observed in both UK and SA hazardous student drinkers. Two major intervention effects obtained in the previous UK study on increased self-efficacy of control over negative affect drinking and over consumption quantity anticipated in the next six months were not replicated in the current study. Instead, the intervention had effects on reducing the belief of drinking to cope with negative affect. These discrepancies observed between the UK and the current study might be attributed to random errors that interfere with the observation of clear and robust intervention effects (Loken & Gelman, 2017), such as variability in small sample sizes, time differences in outcome measurement, differences in sample characteristics that were not controlled for between these two studies (e.g., race, geographical location, use of other substances and etc), and low internal reliability of the outcome measures. It is theoretically possible that intervention effects might differ when measured immediately following the training (UK study) versus across a prolonged period (current study) when the intervention has had greater opportunity to become established in the participants repertoire. As discussed in the previous UK study, drinking to cope may incrementally decline with extended practice of the imagery technique across a longer follow-up period. However, this would better be confirmed via repeated testing every day during the follow-up period to increase the confidence of detecting true intervention effect, as demonstrated in one recent trial study using ecological momentary assessment to show a decline in drinking to cope across six weeks (Blevins et al., 2021). Although not a perfect replication, this proof-of-concept negative affect focused FIT intervention provides some confidence in the delivery of the active intervention to other cross-cultural populations and over an extended follow-up period. Moreover, some valuable lessons can be taken from this FIT trial in SA. For instance, considering limited access to internet/technology accessories in low-and-middle income countries, future studies could consider providing participants with smart phones/watches for delivery and assessment of the intervention. Although online delivery allows certain flexibility for participants to complete follow-up stages, future studies could follow the current findings and consider reducing participants’ burdens by shortening the list of outcome measures to reduce the dropout rate.

The current findings add to the existing literature that imagery retrieval training approach has utility in improving substance use outcomes (Andrade et al., 2012, May et al., 2015). As mentioned in the introduction, another study in our lab has demonstrated that the same FIT intervention abolished noise stress-induced alcohol choices in the lab, suggesting an immediate protective effect of FIT on alcohol-seeking driven by negative affect (Bakou et al., 2021). Similarly, other imagery-based interventions, such as EFT, has also shown effectiveness in reducing alcohol demand in college students in a lab setting (Bulley & Gullo, 2017) and in alcohol dependent individuals at one-week follow-up (Patel & Amlung, 2020). Furthermore, the observed intervention effect on reduced depressive symptoms corroborates with the previous studies on imagery-based interventions improving mental health outcomes. EFT intervention, which train individuals to imagine their best possible self, has shown effective in improving positive mood and affect, and reducing dysfunctional attitudes that are typically presented and maintained in depression (e.g., learned helplessness - interpreting negative events as internal, stable and global, Abramson et al., 1978) (Renner et al., 2014). More support comes from a recent *meta*-analytical study which included 63 experimental studies that applied the best possible self-imagination task, future worry induction, and episodic future simulation, and concluded that imagining the future has a moderate to strong impact on improving affective well-being. Additionally, imagining personal future events has been found to have a stronger influence on affect relative to remembering the past (Schubert et al., 2020). Finally, a similar FIT intervention study has shown a moderate effect in reducing self-harm frequency in young people at 3 months (*d* = 0.65) and a small effect in maintaining improvements from 3 to 6 months (*d* = 0.05), suggesting feasibility of FIT in improving mental health outcomes (Di Simplicio et al., 2020).

It remains unclear what mechanisms might underlie the effects of the current FIT intervention on alcohol-related outcomes. The finding on the correlation between reduced depression and reduced drinking to cope over four weeks may suggest one potential mechanism being building resilience to negative affect. Such growth in resilience to negative affect has been seen with abovementioned imagery-based interventions showing effectiveness in improving affective well-being and also with other behavioural interventions for substance use problems, including personalised intervention on drinking motives (Blevins & Stephens, 2016) and mindfulness-based interventions (Bowen et al., 2014, Grow et al., 2015, Shuai et al., 2020, Stasiewicz et al., 2018, Witkiewitz et al., 2013, Witkiewitz et al., 2005). Specifically, one component of this FIT intervention, implementation intentions, might have contributed to building resilience to negative affect, that is, through the formal rule to respond to negative affect by retrieving an adaptive strategy. Implementation intention studies have demonstrated that setting clear risk-response rules can attenuate affective reactivity (Webb et al., 2012) and improve behaviour change outcomes (Arden and Armitage, 2012, Conner and Higgins, 2010, Knäuper et al., 2009, Norman et al., 2018, Norman and Wrona-Clarke, 2016, Oettingen et al., 2000). The current finding of reduced depressive symptoms associated with reduced drinking to cope may suggest a similar mechanism on building resilience to negative affect. Nevertheless, to allow for a mediational analysis to confirm this possible mechanism of FIT intervention, future studies could consider employing an additional timepoint to assess the effects on affective resilience (using more sensitive assays of mood/resilience as opposed to psychiatric symptom severity) before the effects on negative affect related drinking outcomes (Nock, 2007).

Several limitations of the current design are noteworthy. First, there was no significant reduction in alcohol consumption at follow-up in ether the original UK sample or the current SA replication. These null results could be true negatives but given the small sample sizes and broad scope for methodological limitations, they may be false negatives, and an effect of FIT on alcohol consumption may be detected in other conditions. It is noteworthy that the extended four-week follow-up period in the current study was designed to examine the long terms effect of the FIT intervention, in the hope that group effects in alcohol consumption would emerge at this later timepoint. As no such effect was observed, it is worth reflecting on what design elements might be optimal for detecting such an effect. In previous FIT studies, intervention effects were found on healthy eating behaviour and weight loss at follow-up in people with obesity who wanted to lose weight (Andrade et al., 2016, Solbrig et al., 2019). It is potentially relevant that in these designs, participants were trained to retrieve an image of the benefits of food abstinence, which may compete more directly with desire to eat. By contrast, in our design, participants retrieved an image of an adaptive negative affect coping strategy, which may not compete so directly with consumption behaviour. A similar observation can be made from two recent EFT interventions, where participants were instructed to retrieve images of future goals that directly competed with drinking and found effects on alcohol consumption in college students (Voss et al., 2022) and in alcohol dependent individuals (Athamneh et al., 2022). Thus, it may be important to incorporate personally relevant goals competing with consumption to produce effects on alcohol consumption, while retrieval of adaptive negative affect coping strategies might be better suited to produce changes in depression and coping motives as observed in the current study. The implication is that future FIT studies should combine training of both retrieval practices, in the hope of producing superior therapeutic outcomes.

Another limitation is the high dropout rate after the allocation into intervention groups. Approximately half of the participants in each group, who completed baseline measures, did not engage with the intervention video sufficiently to proceed. Similar dropout rates at the intervention stage were also seen in the previous UK study (Shuai et al., 2021). One possible reason could be that both intervention videos started with health risk information on drinking, which might have resulted in low message acceptance and in turn, cessation of watching the full videos. The feedback from participants who completed the follow-up period showed positive attitudes towards the FIT technique and willingness to continue practising this technique, suggesting an overall good acceptability of FIT, at least amongst those who continued to participate. Future studies might consider evaluating and improving the acceptability of the intervention stage to reduce the dropout and thus, enhance confidence in the generalisability of the approach.

Related to the limitation above, the current study included a relatively small, highly selected student sample, reducing confidence in the generalisability of the findings and replicability in community or clinical samples who differ in a wide range of characteristics (Tackett et al., 2017). Regarding clinical significance of the effects, the active group showed a numerically small reduction in depressive scores, which on average started slightly above the cut-off point for moderate depression of 10 at baseline (13.12) and ended up slightly below at follow-up (9.72) – a change of 3.4 on a 24-point scale. This improvement is below the recommended minimal clinically significant difference of 9 points or 50 % reduction (McMillan et al., 2010) and so we cannot conclude that this improvement is clinically significant. However, the current work is better regarded as an experimental study testing the impact of the specific cognitive manipulation on intermediate outcome measures collected at a longer follow-up timepoint (than before), in an understudied South Africa sample. The manipulation described here is unlikely to be implemented as a complete intervention in itself, but the principles of the general approach might be adopted, developed, and incorporated into a clinical intervention which are themselves tested for efficacy in larger samples of cross-cultural groups. Together, the previous UK study (N = 52) and other FIT studies (N < 50) have shown effects on improving alcohol-related outcomes (Shuai et al., 2021), snacking (Andrade et al., 2016) and self-harm (Di Simplicio et al., 2020), so the current study is consistent with these in suggesting the approach should be tested for therapeutic utility. However, these studies are all pilot trials with lower statistical power than full sized trials, and a full scale trial is required, potentially using the optimal training protocol as noted above, to justify deployment of the approach in a clinical setting (Tackett et al., 2019).

The fourth limitation of the current study is lack of statistical power to detect statistically significant results after the Bonferroni correction was applied for multiple testing, which raises the concern that the reported group by timepoint interactions were due to chance. This concern is compounded by the intervention producing only one overlapping effect (reduced drinking motives for socialising) and some divergent effects (UK - increased self-efficacy of control over negative affect drinking and over consumption quantity; SA - reduced depressive symptoms and drinking to cope) between the previous UK and current SA replication study. Concerns about false positives would have been reduced if all effects had corresponded in both replications. The upshot is that another (larger) replication is needed to confirm the stability of the effects.

Finally, our sample was largely female, which arose from using a convenience sampling approach, so it remains to be seen if the same intervention effects would be obtained in males. It is noteworthy that a previous related study reported effects on reduced alcohol consumption and drunkenness only in females (Wurdak et al., 2016). This raises the concern that FIT might also have this gender bias. To address this, future studies should ensure equal numbers of each gender are established within the final analytic sample after dropout to explore potential gender differences. Despite these limitations, in the content of rising demand for evidence-based brief digital interventions for substance use (Ondersma & Walters, 2020), the current work at least helps justify investment in a larger scale, longer-term trial of negative affect focused FIT with clinical samples to test for effects on recovery, or adolescent samples to test for effects on the trajectory of drinking problems.

In conclusion, online training to respond to negative affect drinking triggers by retrieving adaptive strategies reduced depressive symptoms, drinking to cope with negative affect and for social reasons at four-week follow-up, in hazardous, negative affect student drinkers in SA. These findings converge with previously obtained therapeutic effects of FIT, and support the broader idea that adaptive goal-oriented imaginal training may have therapeutic utility (e.g., Solbrig et al., 2019). The current study replicated the previous UK study by showing improved alcohol-related outcomes in an international population and over an extended follow-up period. It remains to be further investigated whether the current FIT training on retrieval of adaptive negative affect coping strategies combined with personally relevant goals competing with drinking, would have effects on actual alcohol use frequency, and what mechanisms may underlie these effects. Although limitations of the small, female dominant sample may have masked benefits and limited the generalisability of the intervention effects, the current findings suggest that the principles of this FIT approach might be adopted, developed, and incorporated into a clinical intervention to test for intervention efficacy, and justify more experimental work on imagery-based affect mitigation strategies for substance use problems.

## Funding

This research was supported by a Medical Research Council award [MC_PC_MR/R019991/1] to Hogarth and Seedat, and by the South African Research Chair in PTSD (SARChI UID 64811) awarded to Soraya Seedat, hosted by Stellenbosch University, funded by the Department of Science and Innovation (DSI), and administered by the National Research Foundation (NRF).

## CRediT authorship contribution statement

**Ruichong Shuai:** Writing – original draft, Methodology, Formal analysis, Data curation, Conceptualization. **Fatima Ahmed-Leitao:** Writing – review & editing, Methodology, Investigation. **Jenny Bloom:** Writing – review & editing, Methodology, Investigation. **Soraya Seedat:** Writing – review & editing, Supervision, Methodology, Funding acquisition, Conceptualization. **Lee Hogarth:** Writing – review & editing, Supervision, Methodology, Funding acquisition, Formal analysis, Conceptualization.

## Declaration of competing interest

The authors declare that they have no known competing financial interests or personal relationships that could have appeared to influence the work reported in this paper.

## Data Availability

Data will be made available on request.
